# Supplementary material for: Increased homicide played a key role in driving Black-White disparities in life expectancy among men during the COVID-19 pandemic
Source: PLoS One. 2024 Aug 21;19(8):e0308105. doi: 10.1371/journal.pone.0308105 (PMC11338436; doi:10.1371/journal.pone.0308105)
Supplement: S1 Data — (DOCX) [file pone.0308105.s001.docx]

**Supporting Information for**

**Increased homicide played a key role in driving Black-White disparities in life expectancy among men during the COVID-19 pandemic**

**Data and Materials Availability**: All data needed to evaluate the conclusions in the paper are present in the paper and/or the Supplementary Materials. Computer code used to produce the study results is available [here](https://www.openicpsr.org/openicpsr/workspace?goToPath=/openicpsr/208088&goToLevel=project) on OpenICPSR The mortality data can be accessed from the NVSS at https://www.cdc.gov/nchs/data_access/vitalstatsonline.htm#Mortality_Multiple and the population estimates are available from SEER at https://seer.cancer.gov/popdata/download.html.

**Life Expectancy Calculation**

We use standard demographic methods to project life expectancy. Drawing on a number of observed deaths and a given population size, we first estimate age specific mortality-rates:

$${}_{n}{m_{x}=}\frac{{}_{n}{D_{x}}}{{}_{n}{N_{x}}}$$

From these age-specific mortality rates, we assume that the age at death within each interval is the midpoint of the interval:

$${}_{n}{a_{x}=}x+\frac{n}{2} for x<100$$

For the open-ended interval, we approximate age at death using the following formula:

$${}_{n}{a_{x}=}\frac{1}{{}_{n}{m_{x}}} for x=100$$

Finally, we estimate the probability of survival to age *x+n* conditional on being alive at age *x* using the following formula:

$${}_{n}{p_{x}=}1-\frac{n*{}_{n}{m_{x}}}{1+\left( n-{}_{n}{a_{x}} \right)*{}_{n}{m_{x}}}$$

Based on these conditional probabilities of survival, the estimated mean number of years lived from birth is calculated for White and Black men, for 2019, 2020 and 2021.

**Age Decomposition**

The primary age-cause decomposition utilized in this paper is the Arrigia method (1). This is a commonly used approach for decomposing which causes of death explain disparities in life expectancy between two groups. The advantage of this approach is it explicitly controls for differences in the age distribution between groups. In effect, this approach standardizes the age distribution between the two groups, allowing for an age-distribution invariant decomposition by causes of death. This method first decomposes inequalities in life expectancy between age groups, defined by the following formula:

$${{}_{n}\Delta}_{x}=\frac{l_{x}^{1}}{l_{0}^{1}}*\left( \frac{{}_{n}{L_{x}^{2}}}{l_{x}^{2}}-\frac{{}_{n}{L_{x}^{1}}}{l_{x}^{1}} \right)+\frac{T_{x+n}^{2}}{l_{0}^{1}}*\left( \frac{l_{x}^{1}}{l_{x}^{2}}-\frac{l_{x+n}^{1}}{l_{x+n}^{2}} \right)$$

Where ${{}_{n}\Delta}_{x}$ represents the difference in life expectancies between groups 1 and 2, attributed to the age-category beginning with age *x* and going up to age *x*+*n*, ${}_{n}{L_{x}^{2}}$ represent the person-years lived between ages *x* and *x*+*n* by individuals in group 2, $l_{x}^{2}$ represents the proportion of the population of group 2 that live to age *x* ($l_{0}^{1}$ is standard demographic notation and refers to the size of the starting population, or the estimated number of births), and $T_{x+n}^{2}$ represents the person-years lived after age *x*+*n* by members of group 2. The left superscript for $l_{x}, {}_{n}{L_{x}}, T_{x}$ denotes the group (1 or 2) that the measure is referring to.

This method works by controlling for age since rates of death and causes of death are patterned differently among different age groups. Since the age composition of White and Black Americans varies substantially, this step is important. In addition to directly controlling for different death rates among different age groups, this age decomposition also controls for the indirect effect mortality has on individuals' persistence into subsequent age categories. Since certain racial groups suffer from greater mortality in earlier age groups, this affects the number of individuals that age into older age groups, which subsequently drives differences in later rates of mortality.

We use the following formulation for the open-ended age interval (age 100+):

$${}_{\infty}{\Delta_{x}}=\frac{l_{x}^{1}}{l_{0}^{1}}*\left( \frac{T_{x}^{2}}{l_{x}^{2}}-\frac{T_{x}^{1}}{l_{x}^{1}} \right)$$

**References**

1. Arriaga, E. E. (1984). “Measuring and explaining the change in life expectancies.” *Demography*, *21*, 83-96.
