# Supplementary material for: Increased homicide played a key role in driving Black-White disparities in life expectancy among men during the COVID-19 pandemic
Source: PLoS One. 2024 Aug 21;19(8):e0308105. doi: 10.1371/journal.pone.0308105 (PMC11338436; doi:10.1371/journal.pone.0308105)
Supplement: S1 Fig — (DOCX) [file pone.0308105.s002.docx]

**Figure S1. Differences in Homicide Death Rates between Black and White Men, 1990-2021.**

*Sources.* CDC Wonder “Compressed Mortality, 1979-1998”, “Underlying Cause of Death, 1999-2021” data.
