# Supplementary material for: Increased homicide played a key role in driving Black-White disparities in life expectancy among men during the COVID-19 pandemic
Source: PLoS One. 2024 Aug 21;19(8):e0308105. doi: 10.1371/journal.pone.0308105 (PMC11338436; doi:10.1371/journal.pone.0308105)
Supplement: S1 Table — (DOCX) [file pone.0308105.s003.docx]

**Table S1. Black-White Male Cause Decomposition by Year**

| Cause | 2019 | 2020 | 2021 |
| --- | --- | --- | --- |
| Heart diseases | -1.14049 | -1.29528 | -1.16054 |
| Cancers | -0.55049 | -0.42032 | -0.36263 |
| Cerebrovascular diseases | -0.35493 | -0.34856 | -0.34132 |
| Chronic lower respiratory diseases | 0.061563 | 0.011678 | 0.033394 |
| Alzheimer's | 0.014116 | -0.00187 | -0.00273 |
| Diabetes | -0.3395 | -0.39892 | -0.35715 |
| Nephritis, Nephrosis, Nephrotic Symptoms | -0.24548 | -0.22294 | -0.20665 |
| Influenza and pneumonia | -0.06375 | -0.10239 | -0.07314 |
| Septicemia | -0.12693 | -0.10815 | -0.11288 |
| HIV/AIDS | -0.14979 | -0.13052 | -0.11987 |
| Other Infectious diseases | -0.03952 | -0.05047 | -0.04564 |
| Homicide | -1.12035 | -1.41326 | -1.3748 |
| Suicide | 0.311512 | 0.238916 | 0.234226 |
| Traffic Accident | -0.1534 | -0.26862 | -0.27321 |
| Accidental Poisoning | 0.002399 | -0.13845 | -0.24689 |
| Other External Causes | -0.0831 | -0.12448 | -0.11779 |
| Perinatal Deaths | -0.34715 | -0.30676 | -0.20621 |
| Congenital Anomalies | -0.04551 | -0.0368 | -0.0079 |
| COVID | 0 | -1.26367 | -0.63185 |
| Other | -0.67547 | -0.77269 | -0.66846 |
