# Supplementary material for: Increased homicide played a key role in driving Black-White disparities in life expectancy among men during the COVID-19 pandemic
Source: PLoS One. 2024 Aug 21;19(8):e0308105. doi: 10.1371/journal.pone.0308105 (PMC11338436; doi:10.1371/journal.pone.0308105)
Supplement: S2 Table — (DOCX) [file pone.0308105.s004.docx]

**Table S2. Variance Decomposition by Race and Year for Men**

| Cause | White men 2019 | Black men 2019 | White men 2020 | Black men 2020 | White men 2021 | Black men 2021 |
| --- | --- | --- | --- | --- | --- | --- |
| Heart diseases | 52.54067 | 64.2248 | 47.68911 | 52.25337 | 46.92217 | 54.96617 |
| Cancers | 34.06545 | 36.9556 | 30.41361 | 29.30395 | 29.2829 | 30.66495 |
| Cerebrovascular diseases | 8.293698 | 13.6667 | 7.78028 | 11.28871 | 7.960958 | 12.40811 |
| Chronic lower respiratory diseases | 7.273124 | 8.509352 | 6.291704 | 6.872356 | 5.762284 | 6.501441 |
| Alzheimer's | 6.066227 | 8.51926 | 5.618816 | 6.921328 | 5.296696 | 7.310605 |
| Diabetes | 5.376103 | 9.192493 | 5.524316 | 9.370526 | 5.353192 | 9.387369 |
| Nephritis, Nephrosis, Nephrotic Symptoms | 3.138781 | 6.610024 | 2.780333 | 5.320853 | 2.934489 | 5.823907 |
| Influenza and pneumonia | 4.281292 | 5.578454 | 3.817478 | 4.841435 | 2.93198 | 4.474825 |
| Septicemia | 2.590349 | 4.702002 | 2.225506 | 3.52064 | 2.286862 | 3.968622 |
| HIV/AIDS | 0.489535 | 3.084597 | 0.468741 | 2.331991 | 0.404117 | 2.193064 |
| Other Infectious diseases | 2.235462 | 2.990161 | 1.83513 | 2.492566 | 1.834919 | 2.665872 |
| Homicide | 4.584842 | 47.48888 | 5.425851 | 54.28676 | 4.809679 | 52.82974 |
| Suicide | 24.74828 | 11.48587 | 22.70119 | 10.57625 | 21.65912 | 10.86814 |
| Traffic Accident | 16.97281 | 17.70979 | 17.07438 | 19.69471 | 16.62628 | 20.34052 |
| Accidental Poisoning | 33.14143 | 18.83044 | 39.75219 | 23.66142 | 39.78802 | 26.72676 |
| Other External Causes | 14.51942 | 17.60985 | 13.71154 | 16.1939 | 13.56546 | 16.33776 |
| Perinatal Deaths | 13.40805 | 35.94326 | 12.09022 | 30.21785 | 11.61715 | 23.56906 |
| Congenital Anomalies | 8.572541 | 10.52404 | 8.00257 | 8.757698 | 7.544565 | 6.989977 |
| COVID | 0 | 0 | 14.52235 | 29.22575 | 23.95538 | 29.25421 |
| Other | 55.9315 | 73.34577 | 54.24489 | 63.10577 | 55.1188 | 66.82968 |
