# Supplementary material for: Increased homicide played a key role in driving Black-White disparities in life expectancy among men during the COVID-19 pandemic
Source: PLoS One. 2024 Aug 21;19(8):e0308105. doi: 10.1371/journal.pone.0308105 (PMC11338436; doi:10.1371/journal.pone.0308105)
Supplement: Supplementary file 5 [file pone.0308105.s005.docx]

Table S3: Cause-grouping and corresponding ICD-10 codes

ICD 10 Cause grouping	ICD10 4-digit code range	Contents of Cause-group (described using ICD10 cause description)
**(1) Chronic diseases**		
Heart diseases	I00-I09.9	Acute rheumatic fever, chronic rheumatic heart disease
	I11-I11.9	Hypertensive heart disease
	I13-I13.9	Hypertensive heart and renal disease
	I20-I25.9	Ischemic heart disease
	I26-I28.9	Pulmonary heart disease and diseases of pulmonary circulation
	I30-I51.9	Other forms of heart disease
Cancers	C00-D48.9	Neoplasms
Cerebrovascular diseases	I60-I69.8	Cerebrovascular diseases
Chronic lower respiratory diseases	J40-J47	Chronic lower respiratory diseases
Alzheimer’s	G30-G30.9	Alzheimer’s disease
Diabetes	E10-E14.9	Diabetes mellitus
Nephritis, Nephrosis, Nephrotic Symptom	N00-N07.9	Nephritis, Nephrosis, Nephrotic Symptom
	N17-N19	
	N25-N27.9	
**(2) Communicable diseases**		
COVID-19	U07.1	COVID-19
Influenza and pneumonia	J10-J18.9	Influenza and pneumonia
Septicemia	A40-A41.9	Streptococcal and other septicemia
HIV/AIDS	B20-B24.9	HIV disease
Other Infectious Diseases	A00-B99	Certain infectious and parasitic diseases, with the exception of septicemia and STD/NTD
**(3) External causes**		
Homicide	X85-Y09.9	Assault
Suicide	X60-X84.9	Suicide
	X60-X69	Intentional self-poisoning by drugs and alcohol
	X70-X78	Intentional self-harm by suffocation and by objects
	X80-X82	Intentional self-harm by jumping or lying before moving objects
	X83-X84.9	Intentional self-harm by other specified/unspecified means

Traffic Accidents	V02-V04.9<br>V09-V09.9<br>V12-V14.9<br>V19-V19.9<br>V20-V79.9<br>V80-V89.9	Pedestrian injured in other and unspecified transport accidents<br>Pedal cyclist injured in accident with motor vehicle<br>Pedal cyclist injured in unspecified transport/traffic accident<br>Motor-cyclist, occupant of three-wheeled vehicle, car occupant, occupant of pick-up truck, occupant of heavy transport vehicle, or bus occupant injured in transport accident<br>Other land transport accidents
Accidental poisoning	X40-X49.9<br>Y10-Y19.9	Accidental poisoning by drugs and alcohol<br>Poisoning by drugs and alcohol, undetermined intent
Other External Causes	V01-V01.9<br>V90-Y89.9 excluding assault	External causes of morbidity and mortality, with exception of assault
**(4) Infant and child conditions**		
Perinatal deaths	P00-P96	Conditions originating in the perinatal period
Congenital Anomalies	Q00-Q99	Conditions originating from congenital absence or malformation
**(5) Aggregated minor causes (not elsewhere classified)**		
NEC – Not elsewhere classified		NA
