# Supplementary material for: Increased homicide played a key role in driving Black-White disparities in life expectancy among men during the COVID-19 pandemic
Source: PLoS One. 2024 Aug 21;19(8):e0308105. doi: 10.1371/journal.pone.0308105 (PMC11338436; doi:10.1371/journal.pone.0308105)
Supplement: Attachment — Submitted filename: Response to Reviewers PLOS ONE - FINAL.docx [file pone.0308105.s006.docx]

**Response memo to Editors and Reviewers**

“Increased homicide played a key role in driving black-white disparities in life expectancy among men during the COVID-19 pandemic”

***PLOS ONE* (PONE-D-23-42765)**

We thank the reviewers and the editors for their careful reading of our manuscript. We took great care to address each of the reviewers’ questions and suggestions and made substantial changes to the text, tables, and figures accordingly. The current version is substantially improved as a result. Some of their comments required only minor modifications to the text and a brief explanation in this memo, while others required significant analytical attention and, in several instances, extensive rewriting.

Before turning to our reviewer-specific responses, we note that the reviewers were convincing in their suggestions about data selection, age categorizations, scope, and presentation of findings. For this reason, we have completely re-worked our analysis using entirely new and more granular level data to address the reviewers’ concerns about infant mortality (Reviewer 1), replication of previous research (Reviewer 2), and the salience of more precise age groupings (also Reviewer 2). Additionally, in line with the suggestions of Reviewer 3, we have narrowed our scope to male mortality and streamlined our figures to avoid overburdening readers.

Taken together, this response memo and the modifications to the manuscript address each of the important issues raised by the reviewers and editors in a comprehensive and detailed fashion. For each reviewer, we first briefly describe their comments (denoted in italics), followed by our response.

**Reviewer 1**

Reviewer 1 was highly supportive of our study but raised two “minor issues” for further improvement.

1. *Was the initial 5-year age category separated into 0-1 and 1-4 age intervals*? Yes, in our previous analysis, these categories were separated. However, in response to this comment and those of Reviewer 2, we now use the individual-level US multiple cause of death files from the National Vital Statistics System division of the National Center for Health Statistics (available at <https://www.cdc.gov/nchs/data_access/vitalstatsonline.htm#Mortality_Multiple>). This is the exact same data used by Aburto and colleagues (2022). The benefits of these data are twofold. First, we can use single-year age intervals, up to age 100, rather than five-year intervals up to 85+. This provides much greater precision in our estimates. Second, these include the full mortality data and thus there are no issues of censoring that can occur using the CDC WONDER database (small cell sizes are omitted from the CDC WONDER requests). Thus, in our new analysis we separate mortality 0-1 from 1-2, and 2-3, etc.
2. *What method do you use to estimate the number of years lived among those who died within each age category*? In line with standard demographic techniques, we use the midpoint for each age category. We detail this point in the methodological description in the revised Supporting Information (SI).

**Reviewer 2**

Reviewer 2 agreed about the broad appeal of our manuscript but raised both major and minor concerns. We address each in turn.

1. *The life expectancy estimates are quite a bit different from what I have seen in other outlets*. We respond in several ways. First, the reviewer raises an important point about comparability. This, along with the comments of Reviewer 1, encouraged us to seek an alternative data source to ensure our approach aligns with analyses that have been vetted in prior research. To this end, we now use the exact same data source as Aburto and colleagues (2022), both the mortality data and the population data. It is for that reason we find substantively identical results for the 2019-2020 comparison. For instance, according to Aburto et al. (2022), the Black-White life expectancy gap for men went from 5 years in 2019 to 7.1 in 2020. In our analysis, we find the gap went from 5 to 7.2. Second, several of the estimates cited by the reviewer are not sex specific and are thus not comparable to our reported results [the Masters et al. (2024) change from 74.48 to 71.55 is race-specific, but not sex-specific; the same is true for the drop from 74.8 to 71.5 in the United States Life Tables, 2020]. Third, links to the underlying data are provided in both the article and the SI, and the replication package inclusive of the code for all calculations will be made available with the published article.

2. *Stopping counts at 85+ biases mortality estimates*. The reviewer is correct, especially when calculating lifespan variability. This comment also motivated us to seek an alternative data source to address this objection. In the new analysis of the individual multiple cause of death files, we now use single-year age intervals, up to age 100, to obviate this concern. This is the same approach to age categorization as Aburto et al. (2022) and our lifespan variability results now align with theirs (Aburto and colleagues also find B-W lifespan variability decreased during the pandemic). We thank the reviewer for stressing these issues as it pushed us to improve the precision and comparability of our analytical approach.

“*Smaller points*”

3. *The sentence in the introductory paragraph about the toll of homicide is a bit suggestive.* We agree and have altered the sentence in the revised manuscript. It now reads “Not only did the disease burden of the COVID-19 virus fall disproportionately on Black Americans, but the cascading disruptions during the pandemic also took a heavy toll on the Black community, including homicide.”
4. *There are meaningful differences between “murder” and “homicide.”* The reviewer is clearly right, and we now use the term “homicide” throughout to avoid conflating these differences.
5. *Take out the word “only” in the sentence ‘For non-Hispanic White men, the increase was only 22%.”* Done.

**Reviewer 3**

Reviewer 3 offered a positive assessment of our writing and analytical methods. They also offer four comments to further improve the article.

1. *Because the authors don’t elaborate further on the broader categorizations, reference to them should be removed from the manuscript.* The reviewer is correct and we have removed this discussion of broader categories from the revised article.

2. *The current format of the tables and figures is confusing.* The reviewer is convincing on this point and we agree that the previous figures were likely to overburden readers. We have taken this comment to heart and completely overhauled the presentation of findings. Most notably, we now focus on presenting the top five drivers of Black-White mortality disparities in 2019 (plus COVID-19), and then make the full results for all 20 causes of death available in the Supporting Information. In our view, this approach balances breadth and depth without straining readers and aligns with the reviewer’s suggestion to avoid presenting many causes that receive little treatment in the writing. Additionally, we no longer present the different comparisons as separate large panels. Rather, we now present each comparison (e.g., 2019 v. 2020; 2019 v. 2021, etc.) as a separate colored bar in the same graph. This avoids the issue of having to wade through multiple graphs before the figure information is presented, an issue correctly flagged by the reviewer with the previous graphs. We also now display the actual Black-White life expectancy gap in years next to the bars in the life expectancy figures so readers can easily interpret the results. Finally, we altered the color scheme so readers can easily distinguish between the within-year results from the longitudinal results.
3. *The results for men are stark and I would support narrowing the scope to include only male mortality.* This is an excellent suggestion, and one we now fully incorporate into the revised paper. In line with the reviewer’s comment, we now focus only on male mortality throughout the article and Supporting Information and have altered the title to reflect this focus (“Increased homicide played a key role in driving black-white disparities in life expectancy **among men** during the COVID-19 pandemic”).
4. *I think the sentence about “recalibrating our understanding” is a bit overstated.* We agree and have removed this sentence from the revised manuscript. We now highlight at pg. 12 that our study identifies homicide as one of the most promising areas for policy interventions aimed at reversing recent racial disparities in longevity.

We hope it is clear to the reviewers that we took each of their suggestions seriously. We made substantial changes to the manuscript to incorporate their thoughtful comments. Indeed, we have conducted a completely new analysis based on methodological recommendations of the reviewers while simultaneously improving the clarity of our contributions. Accordingly, we see the revised manuscript as improved in virtually every respect. The aims are more concise, the contributions are clearer, the methodological approach aligns with prior research, and the presentation of results is sharper. We hope our efforts have done justice to the reviewers’ and editor’s incisive comments.
